# Scalable synthesis of spherical graphite/ZnO composite anodes for high-performance lithium-ion batteries

**DOI:** 10.1039/d5ra09552b

**Published:** 2026-01-13

**Authors:** Thien Tri Vu, Duy Van Lai, Kien Trung Pham, Phong Quang Le, Thanh Huu Le, Hung Tran Nguyen, Trung-Dung Dang, Duong Duc La

**Affiliations:** a Institute of Materials, Biology and Environment 17 Hoang Sam, Nghia Do Hanoi 100000 Vietnam duc.duong.la@gmail.com; b Institute of Materials Science, Vietnam Academy of Science and Technology No. 18 Hoang Quoc Viet Str., Nghia Do Ward Hanoi Vietnam; c School of Chemistry and Life Sciences, Hanoi University of Science and Technology 1 Dai Co Viet Hanoi 10000 Vietnam

## Abstract

Reimagining graphite anodes through hybrid nano-architectures offers a powerful route to break the long-standing trade-off between capacity and stability in lithium-ion batteries. Here, we design a porous spherical graphite/ZnO (SG/ZnO) hybrid anode *via* a scalable one-pot hydrothermal synthesis combined with high-energy ball milling and mild annealing. The resulting hierarchical framework features robust SG–ZnO interfacial coupling, merging the conductivity and structural resilience of spherical graphite with the high capacity and surface reactivity of ZnO nanosheets. This architecture ensures efficient Li^+^ transport, accommodates volume changes, and suppresses mechanical degradation. The optimized SG/ZnO composite (SG-7/ZnO) delivers a reversible capacity of 423 mAh g^−1^ at 160 mAh g^−1^ over 150 cycles, significantly outperforming pristine SG and ZnO, owing to its excellent charge transport capability and enhanced electrochemical kinetics. This simple yet versatile strategy opens a new pathway for engineering high-performance oxide–carbon hybrids for next-generation rechargeable batteries.

## Abstract 2

A scalable one-pot hydrothermal method integrates porous ZnO nanosheets onto spherical graphite, creating SG/ZnO hybrid anodes with improved conductivity, stability, and lithium storage for high-performance lithium-ion batteries.

## Graphical abstract

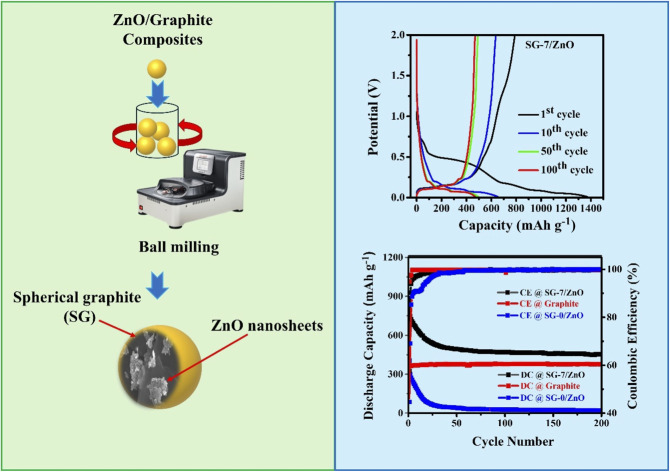

## 1. Introduction

The global shift toward renewable energy sources, such as solar and wind, along with the rapid growth of electric vehicles (EVs) and smart electronics, has created an urgent demand for advanced energy storage technologies that combine high energy density, long cycle life, and safety.^1^ Lithium-ion batteries (LIBs), owing to their superior electrochemical performance, have become the dominant energy storage system in a wide range of applications from portable electronics to grid-scale storage and electric mobility.^2,3^ Graphite has long dominated commercial LIB anodes owing to its stability and safety; however, its limited theoretical capacity (372 mAh g^−1^) and poor performance under low-temperature conditions restrict further improvements in energy density and fast-charging capability. These intrinsic drawbacks, including sluggish Li^+^ diffusion, unstable SEI formation, and lithium plating that induces dendrite growth, highlight the urgent need for alternative anode materials for next-generation energy storage.^4–8^

In the context of rising global energy demand, the search for novel anode materials with high capacity and good stability has become a central research focus to overcome the intrinsic limitations of commercial graphite (372 mAh g^−1^).^9–11^ Various systems have been proposed, among which silicon (Si) stands out owing to its exceptionally high theoretical capacity (∼4200 mAh g^−1^). However, the severe volume expansion (∼300%) and poor electrical conductivity of Si cause rapid performance degradation during cycling.^12–15^ In addition, transition metal phosphides, particularly nickel phosphides (Ni_*x*_P), have recently emerged as competitive conversion-type anodes, delivering higher reversible capacities than graphite.^16^ Despite these advantages, their electrochemical performance remains strongly dependent on synthesis strategies and processing parameters, posing challenges for scalable fabrication. These drawbacks have directed increasing attention toward transition metal oxides (TMOs), which not only offer considerable lithium storage capacity but also provide mechanical robustness and flexible integration with conductive matrices.^17^

Among TMOs, zinc oxide (ZnO) has been extensively investigated as a promising anode candidate for lithium-ion batteries (LIBs) owing to its high theoretical capacity (978 mAh g^−1^), natural abundance, and environmental benignity.^18^ Recent studies highlight that tailoring ZnO into nanostructures such as nanorods, nanowires, nanosheets, and nanoflowers can significantly enlarge the surface area and optimize electronic properties, thereby enhancing storage capacity and cycling performance compared to bulk counterparts. Moreover, the advantages of facile synthesis, low cost, and notable performance improvement through nanoscale design further position ZnO as a highly attractive candidate for next-generation high-performance anodes in lithium-ion batteries.^18,19^ However, its practical application remains hindered by inherent drawbacks, such as severe volume variation during lithiation/delithiation and intrinsically low electrical conductivity, which typically result in rapid capacity fading and poor cycling stability.^20^ To overcome these limitations, numerous strategies have been proposed, among which the development of rationally designed ZnO/carbon composites has emerged as a particularly effective approach.^21,22^

One notable strategy involves constructing hollow, porous, or core–shell architectures in which ZnO is encapsulated or uniformly dispersed within a conductive carbon framework. These designs not only take advantage of the high lithium storage capability of ZnO but also mitigate mechanical stress and enhance electron transport. For example, Song *et al.* reported the synthesis of hollow porous ZnO/C nanocages derived from MOF-5 through a one-step pyrolysis process, achieving a high specific surface area of 256 m^2^ g^−1^ and a carbon content of ∼50 wt%.^23^ Benefiting from the conductive hollow structure, the composite exhibited outstanding lithium storage performance with improved electrolyte infiltration and enhanced mechanical stability. Similarly, Li *et al.* developed core–shell ZnO@C composites *via* chemical vapor deposition (CVD), in which ZnO nanoparticles were encapsulated by N-doped multilayer graphene.^24^ This material delivered a reversible capacity of 390 mAh g^−1^ after 200 cycles at 0.25 A g^−1^ and 204.6 mAh g^−1^ at 1 A g^−1^, attributed to the synergistic effects of high electrical conductivity, enlarged surface activity, and sufficient buffering space for volume expansion. These results highlight the effectiveness of conductive coating and rational nanoarchitecture design in enhancing the electrochemical performance of ZnO-based anodes.

Beyond hollow and core–shell structures, integrating ZnO with various carbon matrices has also attracted considerable attention. Among them, spherical graphite (SG) stands out due to its high packing density, isotropic morphology, excellent conductivity, and established commercial use in LIBs.^25^ The incorporation of ZnO into SG not only enhances the overall lithium storage capacity but also leverages the robust electron pathways and mechanical strength of graphite. Furthermore, the spherical morphology facilitates the formation of a uniform solid electrolyte interphase (SEI), contributing to improved cycling stability.^26^

Despite significant progress in developing ZnO/carbon composites for LIB anodes, most reported strategies still face limitations such as complex synthesis routes, high cost, and poor scalability. In particular, the non-uniform dispersion of ZnO and the weak interfacial contact with the carbon matrix often result in unstable long-term cycling performance. Thus, there remains a critical need for simple, scalable, and cost-effective approaches to engineer ZnO-based composites with optimized conductivity, mechanical buffering, and lithium storage capacity. The present work addresses this gap by introducing a novel synthesis strategy to produce porous spherical SG/ZnO composites with enhanced electrochemical performance and promising prospects for practical LIB applications.

## 2. Experimental section

### 2.1. Materials

Zinc sulfate heptahydrate (ZnSO_4_·7H_2_O, ≥99.0%), and urea (CH_4_N_2_O, ≥99.0%) were obtained from Sigma-Aldrich (USA), while spherical graphite powder (99%) was sourced from the Institute of Chemistry and Materials Science (Vietnam).^27^ All reagents were of analytical grade and used as received without further purification.

Materials used for anode fabrication and electrochemical evaluation were supplied by Xiamen Tmax Battery Equipments Ltd, including lithium metal discs (*ϕ* = 11 mm, 99.9%), polyvinylidene fluoride (PVDF, *M*_w_ = 600 000, 99.6%), *N*-methyl-2-pyrrolidone (NMP, 99.9%), conductive Super P carbon, and a commercial electrolyte (1 M LiPF_6_ in EC/DMC/DEC, 1 : 1 : 1 by volume). Additional components comprised carbon-coated copper foil (current collector), polypropylene membrane separators (Celgard, *ϕ* = 19 mm, 25 µm thickness), and CR2032-type coin cell hardware.

### 2.2. Synthesis of SG/ZnO composite material

The SG/ZnO composites were synthesized *via* a facile one-step hydrothermal approach with optimized reaction and annealing parameters, adapted from previously reported methods.^28^ Briefly, 3.81 g of ZnSO_4_·7H_2_O was completely dissolved in 50 mL of deionized water under continuous magnetic stirring for 15 min. Subsequently, 1.59 g of urea (CH_4_N_2_O) was gradually added to the solution to adjust the pH to approximately neutral (∼7), followed by additional stirring for 15 min. Then, 1.113 g of spherical graphite (SG) was introduced into the precursor solution and vigorously stirred for 1 h to ensure uniform dispersion.

**Fig. 1:**
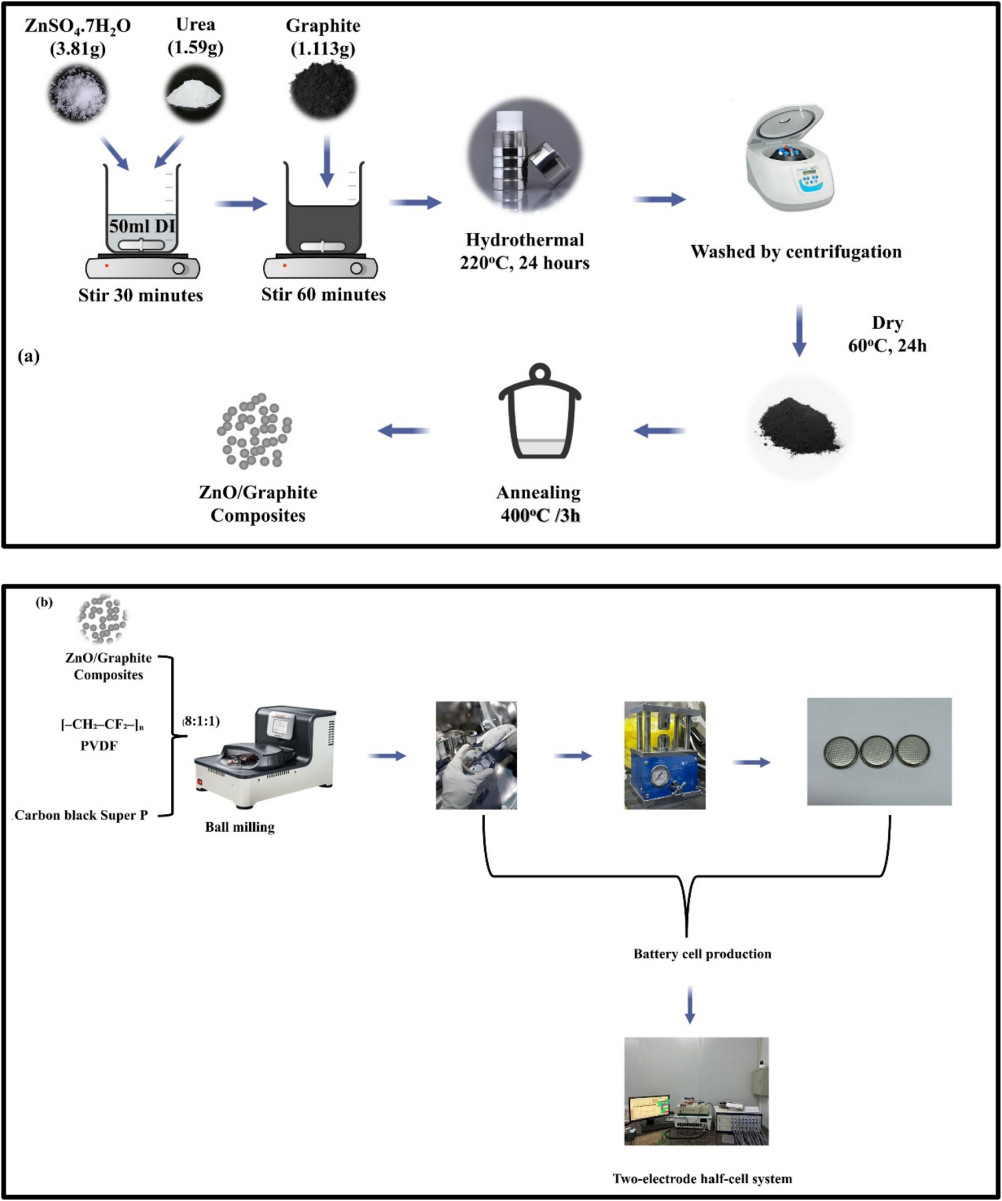
(a) Schematic illustration of the one-pot hydrothermal synthesis process for SG/ZnO composite materials; (b) diagram illustrating battery cell manufacturing.

The resulting homogeneous mixture was transferred to a 100 mL Teflon-lined stainless-steel autoclave and subjected to hydrothermal treatment at 220 °C for 24 h. After natural cooling to room temperature, the obtained black precipitate was collected by centrifugation, thoroughly washed with deionized water and ethanol, and dried in an oven at 60 °C for 24 h. To enhance crystallinity, the dried composite was thermally annealed at 400 °C for 2 h under a controlled gas flow atmosphere. A schematic illustration of the synthesis procedure is presented in Fig. 1.

In addition, a series of SG/ZnO composites with different mass ratios of SG to ZnO (0 : 1, 3 : 1, 5 : 1, 7 : 1, and 9 : 1) were synthesized following the same procedure to investigate the effect of graphite content on the structural and physicochemical properties of the composites. The resulting samples were denoted as SG-0/ZnO, SG-3/ZnO, SG-5/ZnO, SG-7/ZnO, and SG-9/ZnO, respectively.

### 2.3. Physicochemical characterizations

Morphologies of materials are determined by scanning electron microscopy (SEM, Hitachi S-4800). Crystal structures of samples are analyzed by X-ray diffraction (XRD, Bruker D8 Advance) and Raman spectroscopy (Raman microscope, Thermo Scientific DXR3), X-ray photoelectron spectroscopy (XPS/HAXPES). In addition, energy-dispersive X-ray spectroscopy (EDX-mapping) is integrated into the Hitachi S-4800 to determine the chemical composition and uniform distribution of the sample. The specific surface area of the material is determined through Brunauer–Emmett–Teller (BET) analysis using the NOVATouch from Quantachrome.

### 2.4. Electrochemical characterizations

Prior to electrochemical characterizations, prepared composite materials were used as active material to fabricate electrode (Fig. 1b). A mixture of SG/ZnO composite material, PVDF, carbon black Super P with weight ratio of 8 : 1 : 1 was mixed with an adequate amount of NMP solvent with a ball mill until a black homogeneous slurry was obtained. The slurry was then cast onto carbon coated surface of the copper foil substrate with a doctor blade and left for drying at 80 °C in a vacuum oven for 24 hours to obtain the electrode sheet. The sheet was subsequently cut into circular electrodes with a diameter of 10 mm. Each electrode is weighed on an analytical balance to determine the actual mass of active material, which was used to calculate current density and specific capacity for electrochemical tests. These electrodes were assembled into CR2032 coin cells. Tested electrodes were controlled to have active material mass loading of 1 to 2 mg cm^−2^. The electrochemical properties were evaluated using cyclic voltammetry (CV) at a scan rate of 0.05 mV s^−1^, galvanostatic charge–discharge (GCD) at a current density of 100 mAh g^−1^, and electrochemical impedance spectroscopy (EIS) using a WonATech WBCS 3000 system (WonATech, Seoul, Republic of Korea).

## 3. Results and discussion

### 3.1. Morphology characterizations

**Fig. 2:**
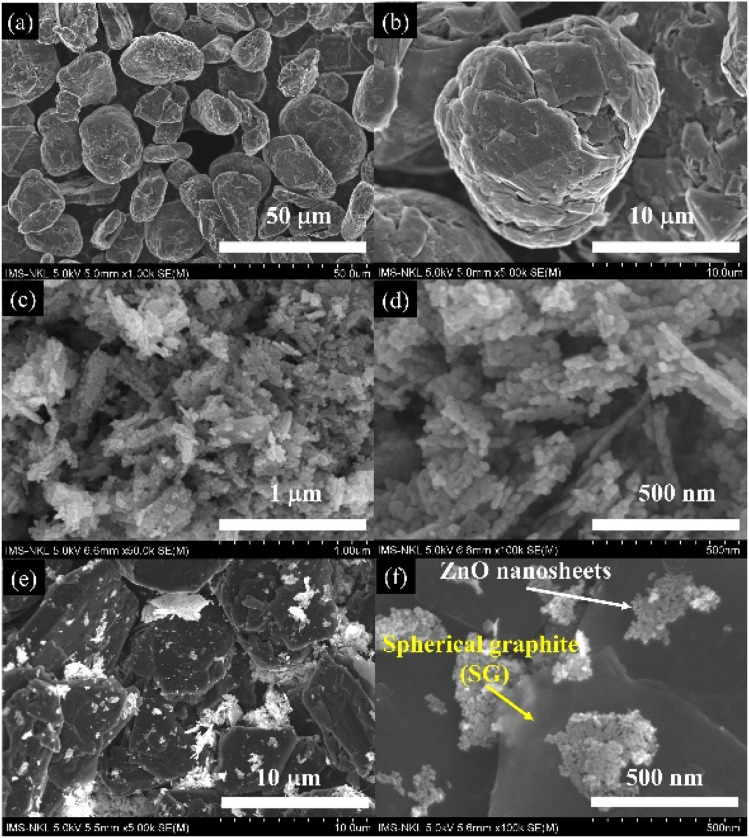
SEM and FESEM images of (a and b) spherical graphite, (c and d) ZnO nanosheets, and (e and f) SG-7/ZnO nanocomposite annealed at 400 °C.

Fig. 2a–f displays the FE-SEM images of purified spherical graphite (SG), hydrothermally synthesized ZnO nanosheets, and the resulting SG-7/ZnO nanocomposite. As shown in Fig. 1a and b, the SG particles adopted from a previous study^27^ retain their spherical morphology with a uniform average diameter of approximately 20 µm. Post-acid treatment, the graphite surface exhibits increased roughness and the presence of pores and surface defects, suggesting effective impurity removal without significant alteration of particle shape or size.

Fig. 2c and d present the morphology of ZnO synthesized *via* hydrothermal processing. The ZnO material primarily exhibits a nanosheet-like structure with a thickness of around 20 nm and pronounced porosity. High-magnification imaging (Fig. 2d) reveals that the sheets are composed of aggregated nanoparticles forming an interconnected, hierarchical network. Such architecture significantly increases the surface area and introduces multiple ion diffusion channels, which are favorable for electrochemical performance enhancement, particularly in lithium-ion storage systems.^29^

Fig. 2e and f illustrate the morphology of the SG-7/ZnO nanocomposite. A limited number of bright ZnO crystallites are observed scattered across the graphite surface. Although the coverage is not extensive, the intimate contact between the porous ZnO domains and the conductive graphite core provides effective electron transport pathways while facilitating lithium-ion diffusion during electrochemical operation.^30^ The combination of high conductivity from SG and large surface area from ZnO is expected to yield a synergistic effect, enhancing charge transfer kinetics and overall electrochemical performance.

**Fig. 3:**
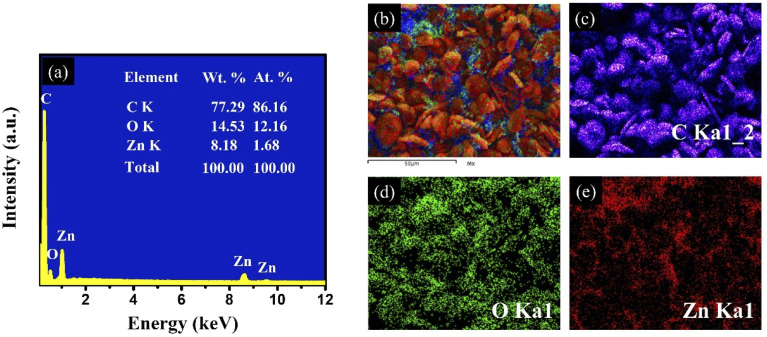
Energy-dispersive X-ray spectroscopy (EDS) elemental mapping of SG-7/ZnO nanocomposite annealed at 400 °C. (a) EDS spectrum, (b–e) elemental mapping of the nanocomposite.

Elemental analysis using energy-dispersive X-ray spectroscopy (EDS) (Fig. 3a) confirms the presence of C, O, and Zn elements in the composite, with atomic percentages of 86.16%, 12.16%, and 1.68%, respectively. The atomic percentage of carbon (86.16 at%) is significantly higher than that of the other elements, confirming that graphite serves as the dominant matrix phase in the composite. The presence of Zn and O in stoichiometric proportions further confirms the incorporation of the ZnO phase within the structure. Furthermore, EDS elemental mapping (Fig. 3b–e) clearly shows that the bright domains observed on the graphite surface correspond to ZnO, thereby verifying the successful deposition and distribution of ZnO on the SG framework.

**Fig. 4:**
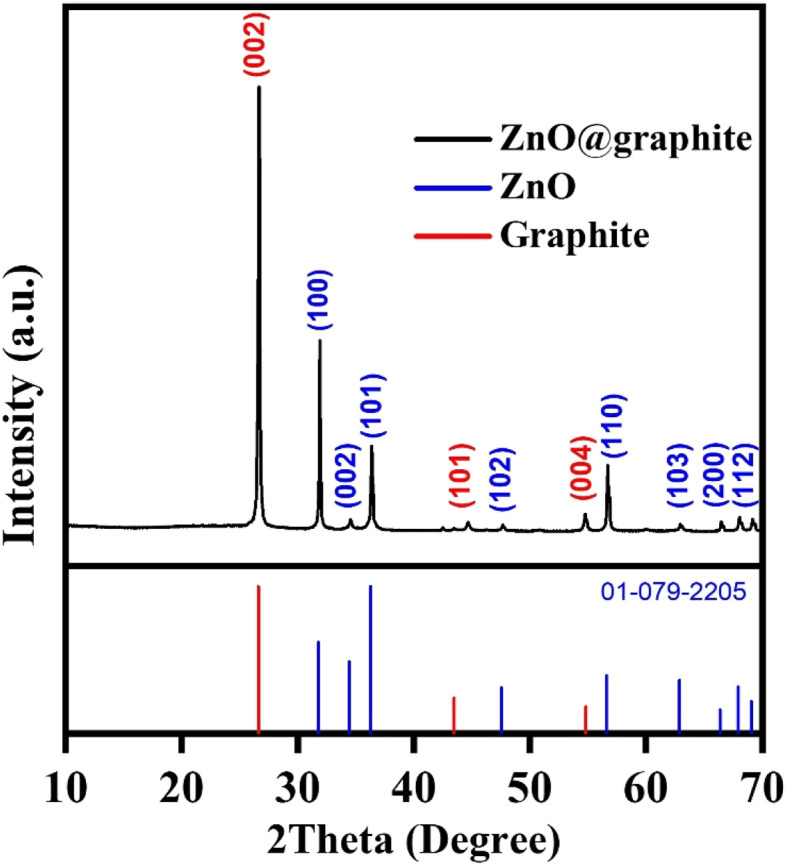
XRD pattern of SG-7/ZnO nanocomposite annealed at 400 °C for 2 h under controlled gas flow.

Fig. 4 displays the X-ray diffraction (XRD) pattern of the SG-7/ZnO nanocomposite annealed at 400 °C for 2 h under a controlled gas atmosphere. The diffraction peaks at 2*θ* = 26.6°, 43.45°, and 54.81° are assigned to the (002), (100), and (004) planes of graphitic carbon, consistent with the characteristic reflections of graphite (JCPDS no. 41-1487).^31^ In addition, the well-resolved peaks corresponding to the (100), (002), (101), (102), (110), (103), (200), (112), and (201) planes confirm the hexagonal wurtzite phase of ZnO (JCPDS no. 36-1451).^28,32,33^ The absence of secondary phases indicates the formation of single-phase ZnO. The sharp and intense reflections suggest high crystallinity. The average crystallite sizes of graphite and ZnO, estimated using the Debye–Scherrer equation,^34^ are approximately 36 nm and 42 nm, respectively within the optimal range for lithium-ion battery applications. The trade-off between crystallinity and Li^+^ diffusion kinetics is a critical aspect for ZnO electrodes. Highly crystalline ZnO provides well-defined diffusion channels, resulting in a high initial reversible capacity. However, repeated lattice expansion/contraction induces microcracks and accelerates capacity fading. In contrast, low-crystallinity ZnO contains disordered domains and voids that can act as strain-relief regions, thus improving cycling stability. On the other hand, the high defect density in amorphous ZnO tends to promote the growth of a thick SEI layer, lowering the initial coulombic efficiency.^35,36^ Consequently, our choice of hydrothermal and post-annealing conditions was aimed at producing ZnO nanosheets with sufficiently high crystallinity to ensure electronic conductivity and Li^+^ mobility, while retaining a porous nanoscale morphology to accommodate volume changes and stabilize long-term performance.

**Fig. 5:**
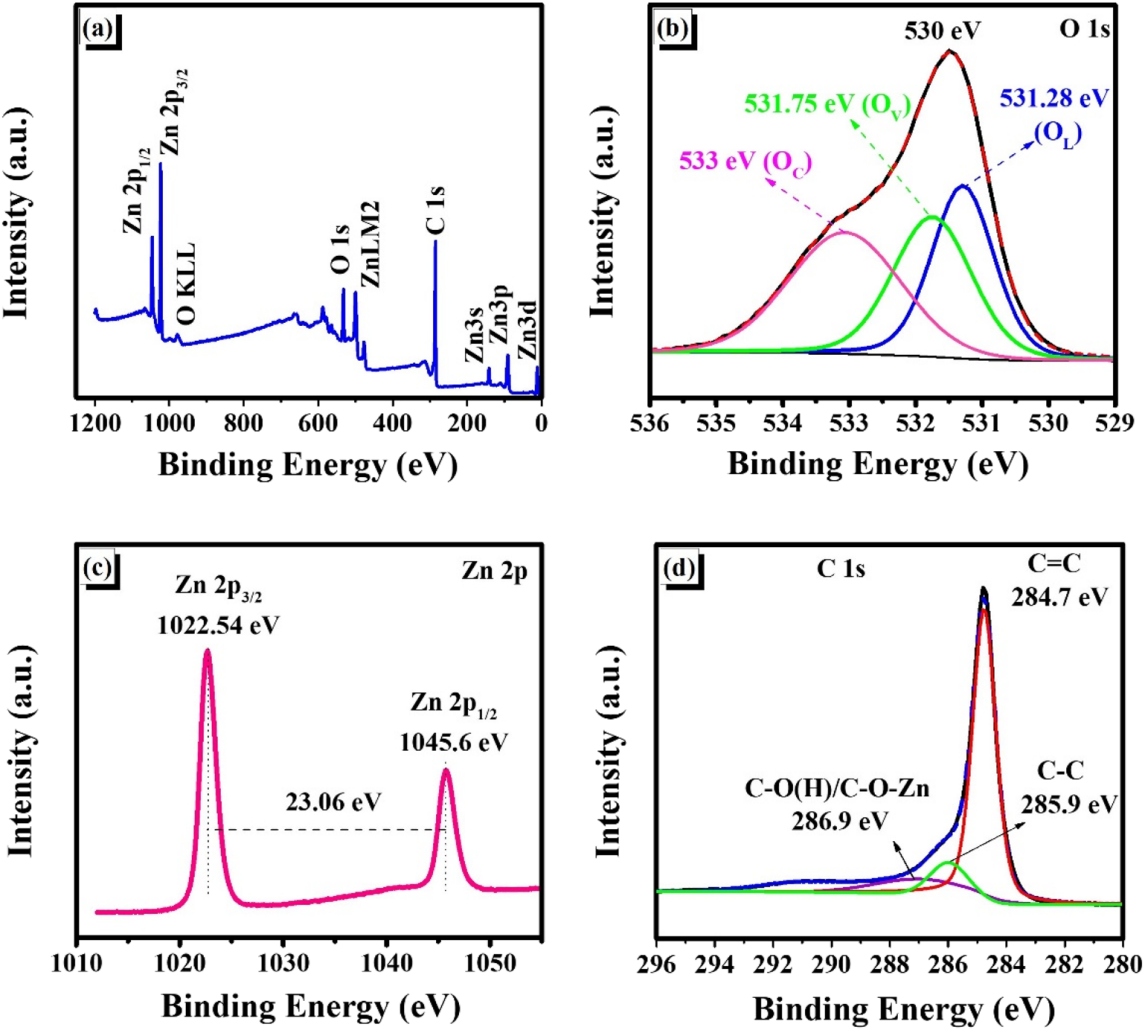
XPS spectra of SG-7/ZnO composite: (a) survey scan; (b) O 1s core level; (c) Zn 2p region; (d) C 1s region.

X-ray photoelectron spectroscopy (XPS) was conducted to examine the elemental composition and chemical states of the SG-7/ZnO composite. The wide-scan spectrum (Fig. 5a) displays distinct signals corresponding to Zn, O, and C, indicating a clean surface without detectable impurities, which confirms the successful integration of ZnO into the composite framework.^18^

The high-resolution O 1s spectrum (Fig. 5b) can be deconvoluted into three components centered at 531.28, 531.75, and 533.3 eV, corresponding to lattice oxygen in ZnO (O–Zn), oxygen vacancies (C=O), and surface carboxylic groups (–COOH), respectively. The presence of surface oxygenated groups, particularly –COOH, is likely related to adsorbed hydroxyl species or residual water from the synthesis process, and they are known to facilitate Li^+^ insertion while suppressing parasitic surface reactions. More importantly, oxygen vacancies not only create an oxygen-deficient Zn–O environment but also induce Zn–O–C bonding at the surface, generating defect sites and Lewis adsorption centers. These features enhance Li^+^ adsorption at the interface, lower the desolvation barrier, and accelerate ion diffusion kinetics. Consequently, improved Li^+^ transport leads to the formation of a thinner and more uniform SEI layer, thereby enhancing reversible capacity and cycling stability. Overall, the synergistic contribution of oxygen vacancies and surface oxygenated groups effectively regulates Li^+^ intercalation dynamics and stabilizes the SEI layer, resulting in superior electrochemical performance of the SG-7/ZnO electrode.^18,37^

The Zn 2p spectrum (Fig. 5c) reveals two well-defined peaks at 1022.54 eV (Zn 2p_3/2_) and 1045.6 eV (Zn 2p_1/2_), with a spin–orbit splitting of 23.06 eV, which is characteristic of Zn^2+^ in the ZnO phase.^38^ This observation confirms the oxidation state of Zn and supports the successful formation of ZnO in the composite.

The C 1s spectrum (Fig. 5d) exhibits three main peaks at 284.7, 285.9, and 286.9 eV, corresponding to sp^2^ C–C, sp^3^ C–C, and oxygenated carbon species (C–O(H)/C–O–Zn), respectively.^18,39^ This indicates the coexistence of graphitic carbon domains and surface functional groups that may affect electrode interfacial properties and electrochemical activity.

**Fig. 6:**
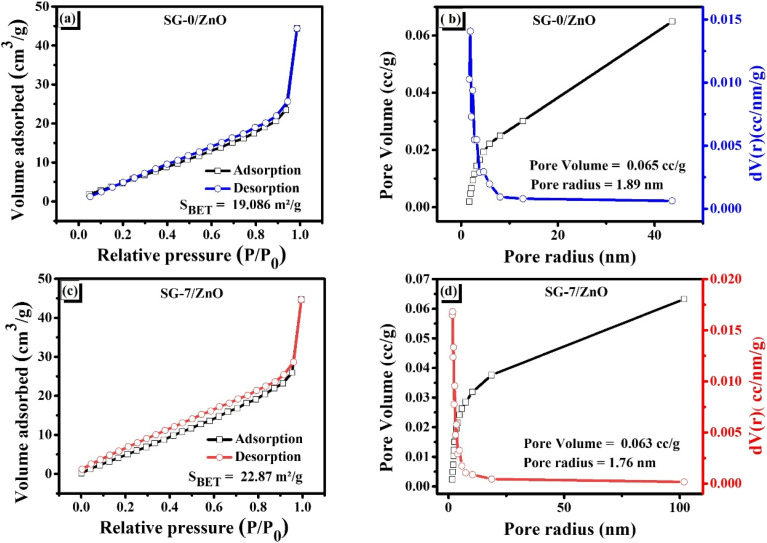
(a and b) Nitrogen adsorption/desorption characterization, and (c and d) pore size and volume of SG-0/ZnO and SG-7/ZnO samples, respectively.

Based on the nitrogen adsorption–desorption isotherms measured at 77 K (Fig. 6a and c), both SG-0/ZnO pristine and SG-7/ZnO nanocomposite samples exhibit characteristic mesoporous behavior, with isotherms of type IV and the presence of an H3-type hysteresis loop, according to IUPAC classification. The occurrence of the H3 loop indicates the presence of slit-like pores, typically associated with partial agglomeration of nanoparticles.^39,40^

Fig. 6a and c presents the nitrogen adsorption–desorption isotherms and pore size distributions of SG-0/ZnO and SG-7/ZnO. Both samples exhibit type IV isotherms with a characteristic H3 hysteresis loop, confirming the presence of mesoporous structures.^41^ The BET surface area of SG-0/ZnO is 19.1 m^2^ g^−1^, whereas SG-7/ZnO shows a nearly twofold increase to 35.4 m^2^ g^−1^. In contrast, the pore volume and average pore diameter decrease slightly from 0.065 cc g^−1^ and 1.89 nm (SG-0/ZnO) to 0.063 cc g^−1^ and 1.76 nm (SG-7/ZnO). Such an increase in surface area accompanied by reduced pore volume and size can be attributed to the partial aggregation and inhomogeneous dispersion of ZnO nanoparticles, which tend to occupy the voids between spherical graphite particles, as also evidenced by SEM observations.

Despite the reduced pore volume, the hierarchical mesopore–macropore structure of SG-7/ZnO provides additional active sites and significantly improves electrode–electrolyte interfacial contact.^42^ This architecture facilitates electrolyte wetting and Li^+^ diffusion pathways within the electrode, while simultaneously accommodating volume variations during lithiation/delithiation.^43–45^ Consequently, the observed pore structure evolution is expected to enhance Li^+^ transport kinetics, contributing to stable cycling performance and improved rate capability.

**Fig. 7:**
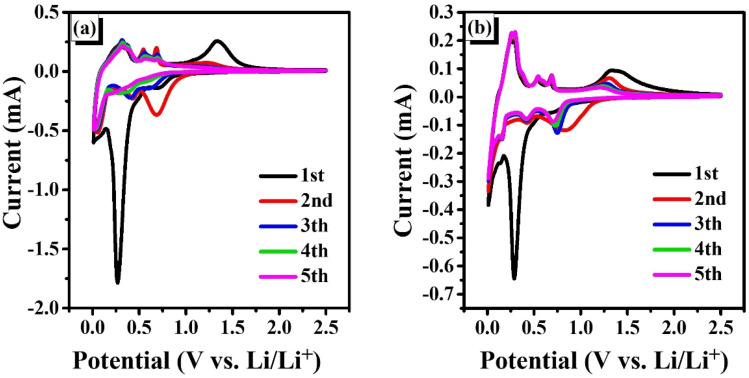
Cyclic voltammetry curves of (a) pristine ZnO and (b) SG-7/ZnO composite electrodes for the first five cycles at 0.1 mV s^−1^ within 0.01–2.5 V *vs.* Li^+^/Li.

Cyclic voltammetry (CV) and electrochemical impedance spectroscopy (EIS) were employed to investigate the formation of the SEI layer, intrinsic resistance, and charge-transfer behavior of the electrodes. Analysis of the CV profiles reveals characteristic redox peaks, providing insights into the Li^+^ insertion/extraction mechanisms and the reversibility of the electrochemical processes. The first five cycles of CV for the ZnO NS and SG-7/ZnO composite electrodes were recorded at a scan rate of 0.1 mV s^−1^ within the potential window of 0.01–2.5 V *vs.* Li^+^/Li (Fig. 7a and b), highlighting differences in reaction kinetics and electrochemical performance between the pristine and composite electrodes.

For the ZnO NS electrode (Fig. 7a), a pronounced cathodic peak at ∼0.26 V was observed in the first cycle, corresponding to the formation of a solid–electrolyte interphase (SEI) layer *via* the irreversible reduction of ZnO with Li^+^ to generate metallic Zn and amorphous Li_2_O [ZnO + 2Li^+^ + 2e^−^ ↔ Zn + Li_2_O; theoretical capacity = 658 mAh g^−1^].^19^ This process contributes to the initial capacity loss but also stabilizes the SEI in subsequent cycles. Additional cathodic features in the range of 0.6–0.8 V and a broad peak near 1.5 V can be attributed to the stepwise conversion of ZnO into metallic Zn and the subsequent alloying of Zn with Li to form Li_*x*_Zn phases.^46^ It should be noted that the theoretical capacity of ZnO (987 mAh g^−1^) arises from the combined contributions of a fully reversible conversion reaction (ZnO → Zn + Li_2_O, 658 mAh g^−1^) and a partially reversible alloying process (Zn + Li ↔ LiZn, 329 mAh g^−1^). While the conversion step dominates the capacity retention during cycling, the alloying step, despite its lower reversibility, still provides a non-negligible contribution to the overall capacity. Therefore, all electrochemical analyses in this work are consistently referenced to the total theoretical capacity of 987 mAh g^−1^.

During the anodic sweep, the peaks located between 0–0.7 V are associated with the dealloying of Li_*x*_Zn back to Zn, while a broad anodic peak centered at ∼1.34 V corresponds to the partial reoxidation of Zn and Li_2_O into ZnO. From the second cycle onward, the CV curves of ZnO NS become nearly overlapped, indicating enhanced reversibility once a stable SEI layer is established.

In contrast, the SG-7/ZnO composite electrode (Fig. 7b) exhibits similar redox features to pure ZnO, confirming that the lithium storage mechanism remains governed by the conversion and alloying reactions of ZnO. However, the intensity and persistence of both cathodic (∼0.26 V) and anodic (∼1.34 V) peaks are markedly improved and remain stable over repeated cycles. The broadened anodic response in the 0.25–1.5 V region suggests overlapping contributions from multi-step dealloying of Li_*x*_Zn and the SG-assisted reversible decomposition of Li_2_O, which is otherwise kinetically hindered in bare ZnO. The progressive increase in anodic current further highlights the catalytic role of SG in facilitating Li extraction and accelerating reaction kinetics.

Overall, while pristine ZnO electrodes rapidly lose their redox activity due to sluggish Li^+^ diffusion and unstable SEI, the SG-7/ZnO composite maintains pronounced and repeatable redox signals, demonstrating improved reversibility and cycle stability. The enhanced electrochemical performance is attributed to the conductive SG framework, which not only promotes electron/ion transport but also stabilizes interfacial reactions, thereby leading to superior cycling durability compared to pure ZnO.^30,32^

**Fig. 8:**
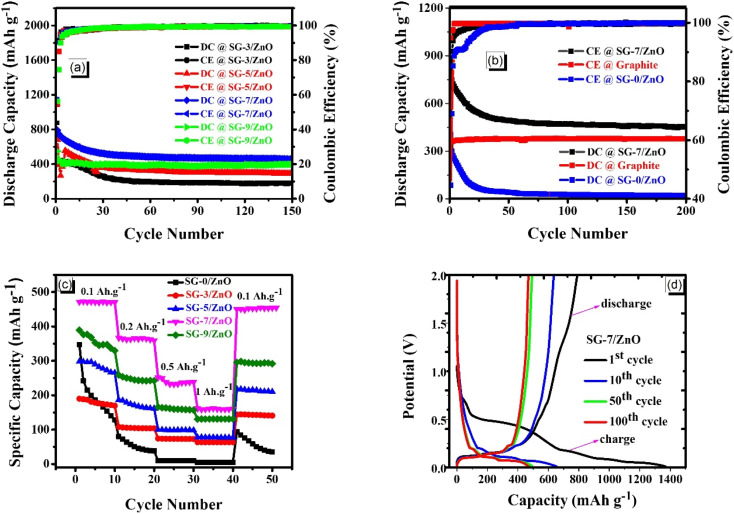
(a) Cycling performance and coulombic efficiency of SG-3/ZnO, SG-5/ZnO, SG-7/ZnO, and SG-9/ZnO electrodes at 0.1 A g^−1^ over 100 cycles. (b) Long-term cycling stability of pristine SG, pristine ZnO, and the SG-7/ZnO composite at 0.1 A g^−1^ over 200 cycles; (c) rate capability of pristine ZnO and SG-*x*/ZnO composite electrodes evaluated at current densities from 0.1 to 1 A g^−1^; (d) charge–discharge curves of the SG-7/ZnO composite at 100 mAh g^−1^ within 0.01–2.0 V.

Fig. 8 clearly illustrates the cycling performance and coulombic efficiency of ZnO-based electrodes with various mixing ratios, along with a comparison between pristine SG, ZnO, and the SG : ZnO composite. Based on the results shown in Fig. 8a, the SG-7/ZnO composite exhibits the most optimal electrochemical performance among the studied ratios. Specifically, the SG-7/ZnO composite sample maintains the highest and most stable specific capacity over 100 cycles, while other compositions show a significant decline in capacity. Notably, the SG-3/ZnO composite sample due to the high ZnO content experiences a rapid capacity fade within the first 30 cycles, similar to the SG-5/ZnO composite sample (red curve). Conversely, the SG-9/ZnO composite sample, with the lowest ZnO content and highest SG content, delivers lower capacity due to the limited amount of active material, despite showing relatively stable coulombic efficiency. The 7 : 1 ratio offers an ideal balance between the two components: a sufficient amount of ZnO to ensure lithium storage capability, and an adequate amount of SG to enhance electrical conductivity, stabilize the structure, and buffer volume expansion during charge/discharge processes.^18,40^ The coulombic efficiency of the SG-7/ZnO composite sample quickly approaches nearly 100% and remains stable throughout the entire cycling test, indicating excellent reaction reversibility. The overall superior performance of this composition highlights the effective synergistic interaction between SG and ZnO, which is maximized at the 7 : 1 ratio.

Fig. 8b compares the cycling behavior of spherical graphite (SG), pristine ZnO, and the SG-7/ZnO composite. The pristine ZnO nanosheet electrode delivers an initial discharge capacity of ∼300 mAh g^−1^ at 0.1 A g^−1^ but shows a low initial coulombic efficiency and a rapid capacity decay during the first 30 cycles.^47^ This behavior is typical of metal–oxide anodes, where the irreversible formation of Li_2_O contributes to capacity loss. After the initial decay, the capacity stabilizes at ∼30 mAh g^−1^ with the coulombic efficiency approaching ∼100%.

In contrast, spherical graphite exhibits a stable initial capacity of ∼360 mAh g^−1^. The SG-7/ZnO composite shows markedly improved cycling stability, maintaining nearly 100% coulombic efficiency and outperforming both individual components over 200 cycles. This synergistic enhancement arises from the improved electronic conductivity and dispersion provided by SG, together with the lithium-storage contribution of ZnO. The nanosheet morphology of ZnO, with particle sizes below 15 nm, further enhances the electrode/electrolyte interfacial contact and improves mechanical robustness during repeated cycling.

All specific capacities are calculated based on the total mass of the composite. To further support the long-term stability of the optimized SG-7/ZnO electrode, extended cycling results are also provided in Fig. S1 (SI).

Fig. 8c compares the rate capability of pristine ZnO and SG-*x*/ZnO composite electrodes at current densities ranging from 0.1 to 1 A g^−1^. The SG-0/ZnO sample (without sphere graphite) delivers the lowest discharge capacity and undergoes rapid fading, indicating poor Li^+^ storage and limited electrochemical stability, likely due to severe volume expansion and the formation of an unstable SEI layer on pure ZnO.^48^ In contrast, all SG-*x*/ZnO composites exhibit significantly improved capacities, with SG-7/ZnO showing the best performance. This sample delivers ∼450 mAh g^−1^ at 0.1 A g^−1^ and retains ∼160 mAh g^−1^ at 1 A g^−1^. Notably, its capacity nearly fully recovers when the current density returns from 1 A g^−1^ to 0.1 A g^−1^, demonstrating excellent structural integrity and reversible Li^+^ insertion/extraction. The stable capacities maintained at each applied current further highlight the outstanding durability and reliable rate performance of the SG-*x*/ZnO composites.

Fig. 8d and S2 (SI) displays the galvanostatic charge–discharge profiles of the SG-*x*/ZnO composite at 100 mAh g^−1^ within 0.01–2.0 V for the 1st, 10th, 50th, and 100th cycles. The initial discharge capacity reaches ∼1350 mAh g^−1^, exceeding the theoretical capacity of ZnO (978 mAh g^−1^). This excess capacity is commonly associated with electrolyte decomposition, Li^+^ consumption, and the formation of the solid electrolyte interphase (SEI), which is also evidenced by the pronounced plateau near 0.5 V in the first discharge. From the 10th cycle onward, the charge–discharge curves overlap more closely, indicating improved electrochemical reversibility after the initial activation. The disappearance of the 0.5 V plateau further confirms that SEI formation predominantly occurs during the first cycle. Even after 150 cycles, the electrode maintains a discharge capacity of ∼450 mAh g^−1^, demonstrating the stable cycling behavior of the SG-7/ZnO composite.

Table 1 summarizes the recent state-of-the-art ZnO/C anodes and compares them with the SG/ZnO composite reported in this work. In general, ZnO/C systems exhibit improved cycling stability and rate capability compared to pristine ZnO, owing to enhanced electrical conductivity and mechanical buffering provided by the carbon phase.

**Table 1:** Comparison of the electrochemical performance of the SG/ZnO electrode with other ZnO/carbon composite anodes reported in recent studies

	Material composition	Synthesis method	Cycling stability (retention/cycles/current density)	Rate performance (mAh g^−1^ at ∼1 A g^−1^)	Ref.
1	ZnO/C	One-step calcination	212 mAh g^−1^/100/0.1 A g^−1^	125 mAh g^−1^	49
2	Porous ZnO@C nanoplates	Ultrasonication	207 mAh g^−1^/100/0.5 A g^−1^	187.3 mAh g^−1^	50
3	ZnO/mesoporous carbon	A facile, scalable precipitation	637 mAh g^−1^/200/0.1 A g^−1^	180 mAh g^−1^	51
4	Carbon fiber@pore-ZnO	Solution-based precipitation	510 mAh g^−1^/300/0.1 A g^−1^	270 mAh g^−1^	52
5	ZnO/rGO	Electrostatic spray deposition (ESD)	360 mAh g^−1^/200/0.2 A g^−1^	197.1 mAh g^−1^	52
6	ZnO/carbon felt (CF)	A facile solvothermal	520.2 mAh g^−1^/100/0.1 A g^−1^	278.6 mAh g^−1^	53
7	2Zn–graphite (2 ALD cycles)	Atomic layer deposition	420 mAh g^−1^/500/0.2C (1C = 372 mA g^−1^)	109 mAh g^−1^ at 5C	18
8	Spherical graphite/ZnO	One-step hydrothermal	423 mAh g^−1^/150/0.1 A g^−1^	160 mAh g^−1^	This work

The SG/ZnO composite delivers a specific capacity of 423 mAh g^−1^ after 150 cycles at 0.1 A g^−1^, which is comparable to or higher than many reported ZnO/C anodes prepared *via* more complex routes (*e.g.*, ZnO/C *via* calcination: 212 mAh g^−1^; porous ZnO@C: 207 mAh g^−1^; ZnO/rGO: 360 mAh g^−1^). Although some highly porous carbon-based structures, such as ZnO/mesoporous carbon or carbon fiber@ZnO, show superior long-term capacity retention, the SG/ZnO composite demonstrates stable performance with a simple, one-step hydrothermal synthesis.

Regarding rate performance, reported ZnO/C anodes typically achieve ∼100–280 mAh g^−1^ at ∼1 A g^−1^. The SG/ZnO electrode reaches 160 mAh g^−1^ at ∼1 A g^−1^, which is within the typical range for similar ZnO/C composites, particularly considering the moderate ZnO loading and the facile synthesis route. This indicates a balanced combination of electrochemical performance and practical scalability.

**Fig. 9:**
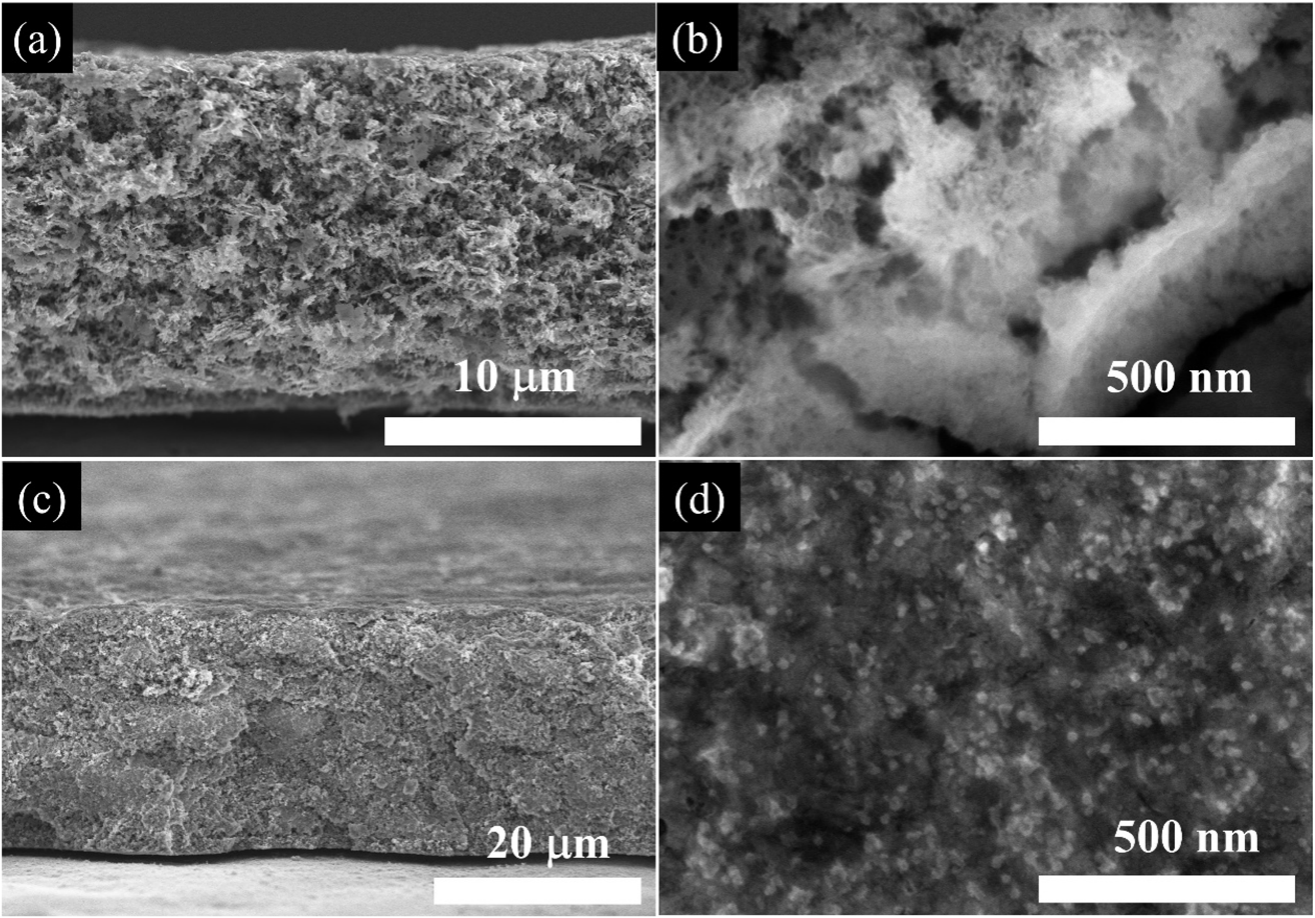
Cross-sectional SEM images of (a and b) pristine ZnO and (c and d) SG/ZnO electrodes: (a and c) fresh electrodes and (b and d) after 200 charge–discharge cycles.

Fig. 9a and c show cross-sectional SEM images of pristine ZnO and SG/ZnO electrodes, respectively, while Fig. 9b and d depict the same electrodes after 200 charge–discharge cycles. The fresh ZnO electrode exhibits a relatively rough surface with loosely packed particles, whereas the SG/ZnO electrode displays a more uniform and compact morphology due to the presence of the SG framework. After 200 cycles, the ZnO electrode shows significant particle agglomeration and surface roughening, indicative of SEI growth and electrode degradation. In contrast, the SG/ZnO electrode retains its original morphology with minor changes, suggesting that the SG framework helps maintain structural integrity and suppress excessive SEI formation. These observations imply improved electrochemical stability and reduced volume expansion in SG/ZnO electrodes during cycling.

**Fig. 10:**
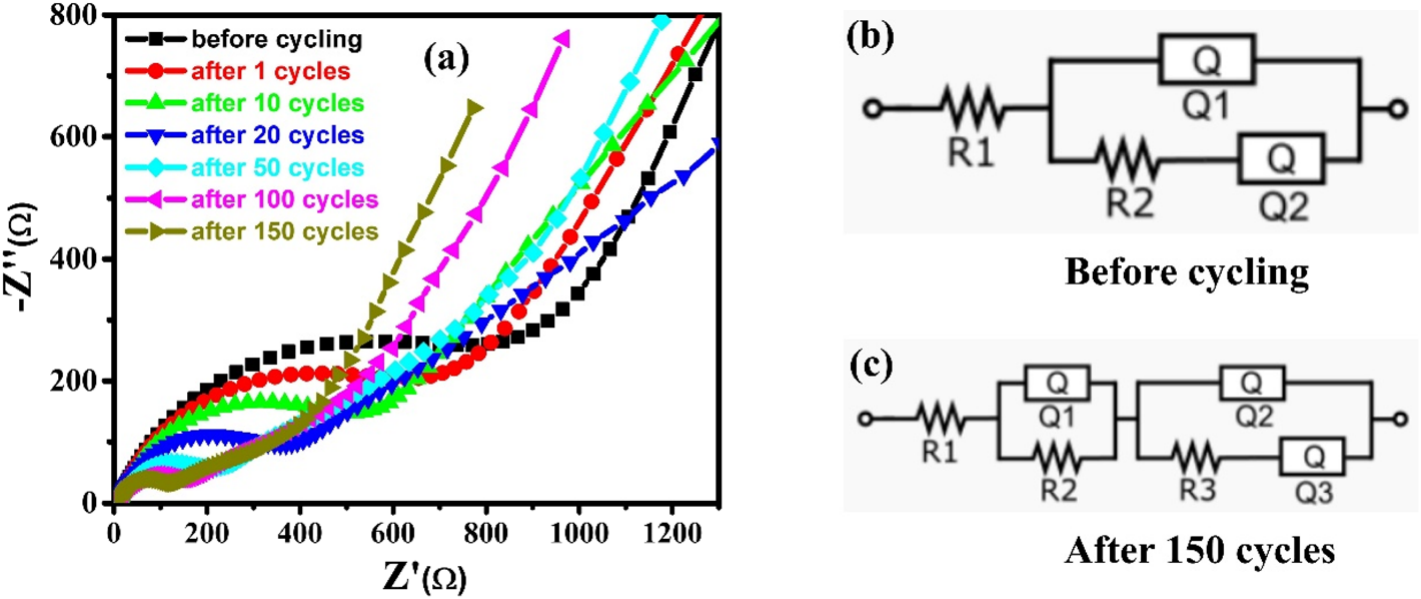
(a) EIS spectra of SG/ZnO electrodes before and after 200 charge–discharge cycles; equivalent circuit models used to fit the spectra (b) before cycling and (c) after cycling.

To elucidate the origin of the excellent battery performance, electrochemical impedance spectroscopy (EIS) of the SG/ZnO electrodes was measured before and after 200 charge–discharge cycles (Fig. 10). The spectra were fitted using the equivalent circuit shown, where *R*_1_ represents the bulk resistance of the cell, *R*_2_/*Q*_2_ correspond to the SEI layer, *R*_3_/*Q*_3_ to the charge-transfer process, and *Q*_3_ accounts for Warburg diffusion.^54,55^ Prior to cycling, only a single semicircle associated with charge-transfer was observed, as the SEI had not yet formed. The electrolyte resistance (*R*_1_) remained nearly unchanged (6.99 Ω → 7.6 Ω), whereas the charge-transfer resistance markedly decreased from 879 Ω to 112 Ω after cycling, indicating significantly enhanced electrochemical activity of the SG/ZnO electrode.

## 4. Conclusions

This work introduces a novel, scalable route to engineer porous spherical graphite/ZnO (SG/ZnO) composite anodes *via* one-pot hydrothermal synthesis combined with pre-milling and mild annealing. Unlike conventional ZnO–carbon composites, the optimized SG-7/ZnO design achieves an ideal balance between electrical conductivity, structural stability, and high lithium storage capacity. The conductive SG framework effectively buffers ZnO volume changes, accelerates charge transport, and stabilizes the SEI, resulting in outstanding cycling stability and rate performance. This simple and tunable synthesis concept offers a versatile platform for designing next-generation conversion-type anodes and can be readily extended to other oxide–carbon architectures for lithium-, sodium-, and potassium-ion batteries.

## Conflicts of interest

There are no conflicts to be declare.

## Supplementary Material

RA-016-D5RA09552B-s001

## Data Availability

Data for this article, including SEM, XRD, BET, FTIR, *etc.*, and electrochemical property measurements are available at Open Science Framework at https://osf.io/u4hp3/. This SI provides additional data on the scalable synthesis of spherical graphite/ZnO composite anodes for high-performance lithium-ion batteries. It includes figures on long-term cycling stability, charge–discharge profiles, and differential capacity curves of the SG/ZnO electrodes. Supplementary information (SI) is available. See DOI: https://doi.org/10.1039/d5ra09552b.
