# Supplementary material for: Scalable synthesis of spherical graphite/ZnO composite anodes for high-performance lithium-ion batteries
Source: RSC Adv. 2026 Jan 13;16(3):2754–66. doi: 10.1039/d5ra09552b (PMC12797207; doi:10.1039/d5ra09552b)
Supplement: RA-016-D5RA09552B-s001 [file RA-016-D5RA09552B-s001.pdf]

This journal is © The Royal Society of Chemistry 2025

Electronic Supplementary

Scalable synthesis of spherical graphite/ZnO composite anodes for high-performance lithium-ion batteries

Thien Tri Vu[1], Duy Van Lai[2], Kien Trung Pham[1], Phong Quang Le[1], Thanh Huu Le[1], Hung Tran Nguyen[1], Trung-Dung Dang[3], Duong Duc La[1,*]

[1]*Institute of Materials, Biology and Environment, 17 Hoang Sam, Nghia Do, Ha Noi, 100000, Vietnam.*

[2] *Institute of Materials Science, Vietnam Academy of Science and Technology, No. 18 Hoang Quoc Viet Str., Nghia Do Ward, Hanoi, Vietnam.*

[3]*School of Chemistry and Life Sciences, Hanoi University of Science and Technology, 1 Dai Co Viet, HN 10000, Vietnam.*

Correspondence: duc.duong.la@gmail.com

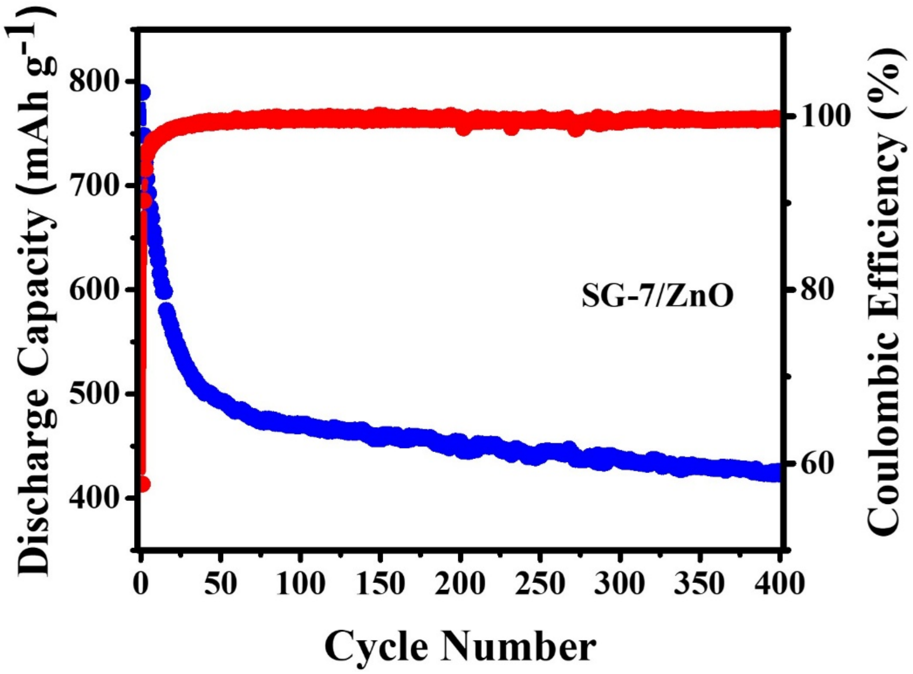

Figure S1. Long-term cycling stability of the SG-7/ZnO composite electrode at a current density of 0.1 A g^{-1} over more than 400 cycles.

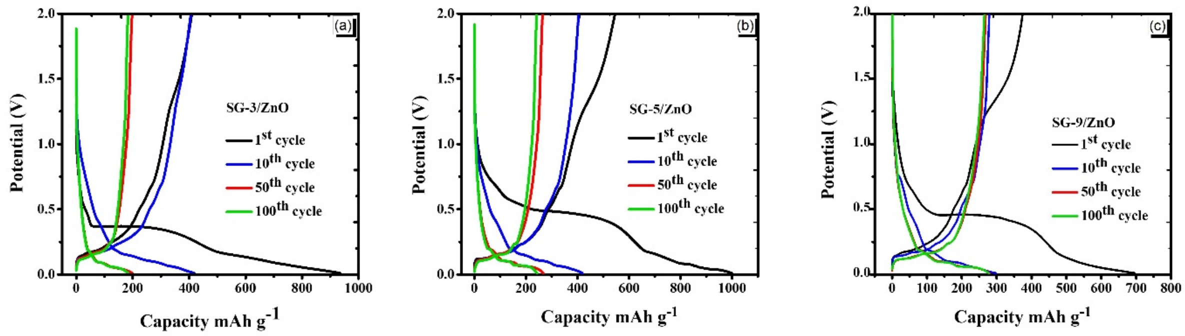

Figure S2. Charge–discharge profiles of SG-x/ZnO electrodes at 100 mAh g⁻¹ (0.01–2.0 V) over selected cycles.

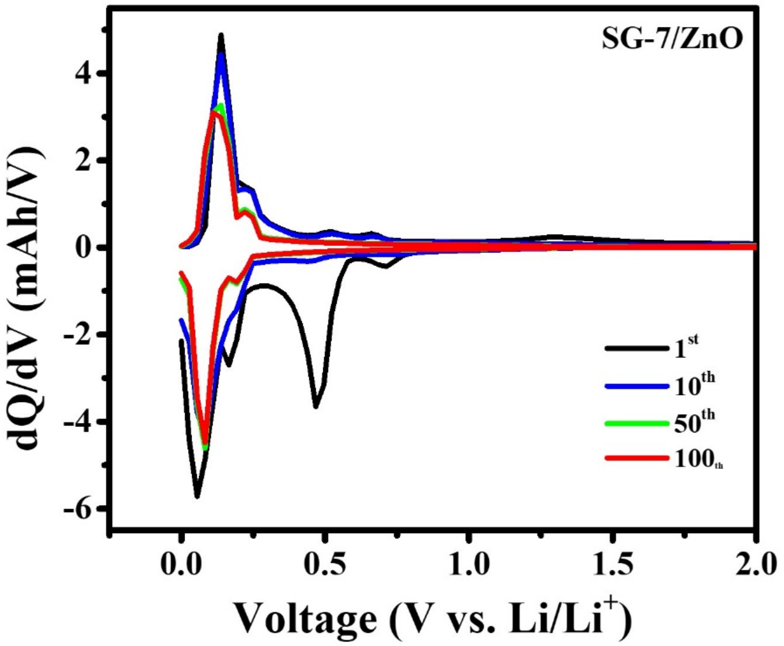

Figure S3. Differential capacity (dQ/dV) curves of SG-7/ZnO electrode at 1st (black), 10th (blue), 50th (green), and 100th (red) cycles within 0.01–2.0 V vs. Li/Li^+.
